# Study of Resistive-Type Superconducting Fault Current Limiters for a Hybrid High Voltage Direct Current System

**DOI:** 10.3390/ma12010026

**Published:** 2018-12-21

**Authors:** Lei Chen, Huiwen He, Guocheng Li, Hongkun Chen, Lei Wang, Xiaoyuan Chen, Xin Tian, Ying Xu, Li Ren, Yuejin Tang

**Affiliations:** 1 School of Electrical Engineering and Automation, Wuhan University, Wuhan 430072, China; li_guo_cheng@163.com (G.L.); chkinsz@163.com (H.C.); 2 State Key Laboratory of Power Grid Environmental Protection, China Electric Power Research Institute, Wuhan 430074, China; husthhw@126.com (H.H.); wanglei8@epri.sgcc.com.cn (L.W.); 3 School of Engineering, Sichuan Normal University, Chengdu 610101, China; chenxy@sicnu.edu.cn; 4 School of Electronic Information, Wuhan University, Wuhan 430072, China; xin.tian@whu.edu.cn; 5 State Key Laboratory of Advanced Electromagnetic Engineering and Technology, Huazhong University of Science and Technology, Wuhan 430074, China; xuying@hust.edu.cn (Y.X.); renli@mail.hust.edu.cn (L.R.); tangyj@mail.hust.edu.cn (Y.T.)

**Keywords:** hybrid high voltage direct current transmission system, resistive-type superconducting fault current limiter, scheme design, short-circuit fault, Yttrium barium copper oxide materials, transient simulation

## Abstract

In this paper, a hybrid high voltage direct current transmission system containing a line commutated converter and a voltage source converter is developed. To enhance the robustness of the hybrid transmission system against direct current short-circuit faults, resistive-type superconducting fault current limiters are applied, and the effectiveness of this approach is assessed. Related mathematical models are built, and the theoretical functions of the proposed approach are expounded. According to the transient simulations in MATLAB software, the results demonstrate that: (i) The superconducting fault current limiter at the voltage source converter station enables to very efficiently mitigate the fault transients, and owns an enhanced current-limiting ability for handling the short-line faults. (ii) The superconducting fault current limiter at the line commutated converter station is able to mildly limit the fault current and alleviate the voltage drop, and its working performance has a low sensitivity to the fault location. At the end of the study, a brief scheme design of the resistive-type superconducting fault current limiters is achieved. In conclusion, the application feasibility of the proposed approach is well confirmed.

## 1. Introduction

In recent years, hybrid high voltage direct voltage (HVDC) technology has received continuously increasing attention, and it is known as an advanced option for long distance as well as large-scale power transmission [1,2]. In principle, a hybrid HVDC transmission system contains a line commutated converter (LCC) and a voltage source converter (VSC). The LCC serves as a rectifier station to save the capital cost, and the VSC acts as an inverter station to strengthen the operational flexibility. Due to integrating the merits of the LCC and VSC, the hybrid HVDC owns the following technical characteristics: (i) inexistence of commutation failure, (ii) enhanced competence to support weak/passive networks, (iii) flexible control of active and reactive power.

For promoting the development of hybrid HVDC technology, scholars have conducted some fundamental researches, which focus on the measure of alternating current (AC) system strength and the small-signal dynamics [3,4]. However, there are little studies on enhancing the robustness of a hybrid HVDC transmission system against DC short-circuit faults. In a sense, the hybrid HVDC may have a complex fault issue, since the DC fault currents of the LCC and VSC stations have essential differences with each other. For the DC fault current of the LCC station, it could be properly adjusted by the firing angle controller, and applying an additional current-limiting solution is able to bring a better fault suppression effect. Concerning the DC fault current of the VSC station, it rises very fast and cannot be removed even though the power electronic switches are blocked, while the anti-parallel diodes act as a freewheeling bridge circuit to feed the fault current [5]. Thus, it becomes an urgent and inevitable requirement to introduce an efficient current-limiting approach in the VSC station.

In this study, our research group suggests using superconducting fault current limiters (SFCLs) to solve the DC fault issue in the hybrid HVDC, and it is because SFCL is a very competitive current-limiting device with excellent performance superiorities, such as automatic trigger and rapid response [6,7,8,9,10]. Based on a comprehensive literature review, Table 1 lists a summary of the studies of SFCLs in different HVDC networks. Technically speaking, the current studies are mainly concerned about a pure LCC-HVDC or VSC-HVDC network.

In [11], a flux-coupling-type SFCL is applied to address the commutation failure in a pure LCC-HVDC grid. In light of different fault types and fault resistances, this SFCL’s impacts on reducing the duration of the commutation failure and accelerating the fault recovery are confirmed. In [12,13], the performance behaviors of the resistive type SFCL on mitigating the commutation failure of a LCC-HVDC grid are studied. Different installation sites of the SFCL for the HVDC network are assessed, and a suitable optimal method of the SFCL resistance is investigated.

In [14,15,16,17], the SFCLs such as resistive-type, saturated iron-core-type, and hybrid-type are selected to inhibit the DC fault current of a pure VSC-HVDC network. A few helpful contributions regarding the parameter optimization and techno-economic evolution of the SFCLs for the VSC-HVDC network protection are obtained. In addition, some scholars preliminarily explore the influences of the resistive and inductive SFCLs on improving the operation reliability [18], and strengthening the fault ride-through (FRT) of wind plants connected to the VSC-HVDC network [19].

To the best of our knowledge, there are no related reports about the systematic application of resistive SFCLs in a hybrid 500 kV HVDC network. When two resistive SFCLs are respectively installed at the LCC and VSC stations to withstand the DC fault, it is crucial to investigate how the two SFCLs can protect a hybrid HVDC network subject to the change of current-limiting parameters, fault resistances, and fault locations. In addition, it is critical to clarify the performance differences between the two SFCLs and lay a foundation for the scheme design of superconducting devices.

Aiming at the aforementioned tasks, this paper is devoted to studying and assessing the application feasibility of resistive SFCLs in a 500 kV hybrid HVDC network. The paper is arranged as follows. Section 2 states the analytical model of the hybrid HVDC including the SFCLs, and discusses the theoretical functions of the SFCLs to the DC fault behaviors. Section 3 conducts the simulation analyses and performance comparison, where different current-limiting parameters, fault severity levels and fault locations are taken into consideration. In Section 4, a brief scheme design of the SFCLs basing YBCO material is given. Section 5 recaps the main conclusions and suggests improvements in the future.

## 2. Theoretical Analysis

### 2.1. Analytical Model of the Hybrid HVDC Including the SFCLs

**Figure 1:**
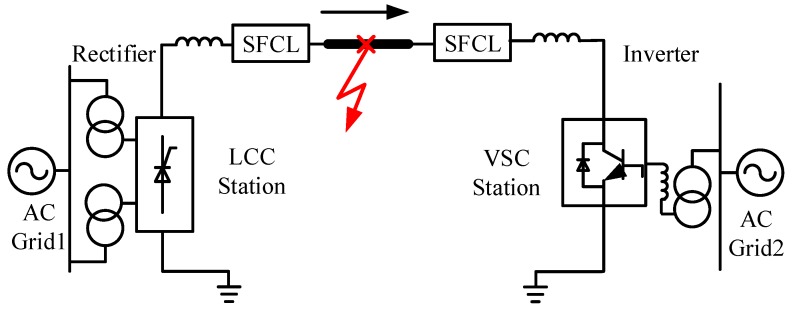
Allocation of the resistive SFCLs in a hybrid HVDC system with the LCC and VSC station.

Figure 1 indicates the schematic connection of the hybrid HVDC system including two resistive SFCLs. The system is a 500 kV bipolar hybrid LCC-VSC HVDC link (only positive pole is denoted here), and the two SFCLs are installed at the LCC station (rectifier side) and the VSC station (inverter side), respectively. For the analytical model of the hybrid HVDC system, this study mainly considers the following factors: (i) The two AC grids are represented by equivalent AC voltage sources with series impedances [20]. (ii) The DC transmission line is represented by an equivalent “resistance-inductance (R-L)” model. (iii) The LCC station adopts a constant DC current control to generate the firing angle, and the VSC station uses a direct current control mode [21]. Detailed modeling information as well as mathematical equations can be found in Appendix A.1, Appendix A.2 and Appendix A.3.

**Figure 2:**
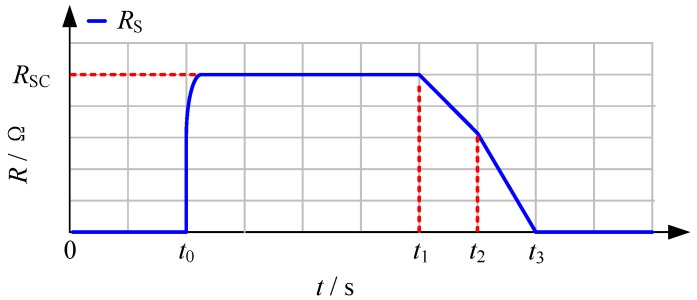
The simplified mathematical model of the resistive SFCL.

Based on the second generation Yttrium barium copper oxide (YBCO) material, Figure 2 describes the equivalent modeling of a resistive SFCL at different time-scales [16,22,23], and the change rule of the SFCL resistance is written as:

(1) R(t)={0(t<t0)RSC[1−exp(−t−t0τ)]12(t0≤t<t1)a1(t−t1)+b1(t1≤t<t2)a2(t−t2)+b2(t2≤t<t3)

where *t* is the time constant; *R_SC_* is denoted as the normal-state resistance of the SFCL. From Equation (1), the SFCL’s equivalent model is explained as: (i) *t*_0_ is the quench-starting time; *t*_1_ is the first-stage recovery-starting time; *t*_2_ is the secondary-stage recovery-starting time; *t*_3_ is the completed recovery time. (ii) *a*_1_, *a*_2_, *b*_1_, and *b*_2_ are expressed as the model coefficients, respectively.

From the literature, the modeling of the two-stage recovery time is mainly based on the experimental studies for superconducting elements, and the temperature effect could be the potential reason. When the SFCL starts to recover to the superconducting state, the accumulated joule heat under the current-limiting operation leads to the temperature rising and make the SFCL resistance have a constrained variation trend, and it is defined as the first-stage recovery process. After the heat is dissipated, the lowering of temperature will make the SFCL resistance have a faster drop to zero, and it is defined as the two-stage recovery process. As for a precise resistive SFCL model on account of the “power-law” equation, this equivalent SFCL model has multiple operational segments, and it is still valid to reflect the transient properties of the resistive SFCL.

### 2.2. Impacts of the SFCLs on the DC Fault Currents

**Figure 3:**
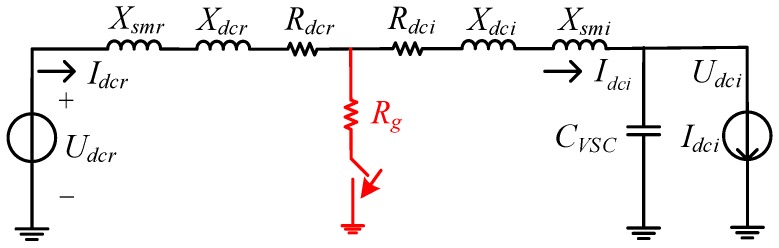
The equivalent circuit of the DC-link.

In this section, the impacts of the SFCLs on the DC fault currents of the LCC and VSC stations are discussed. As shown in Figure 3, it indicates the equivalent circuit of the DC-link. *U_dcr_*, *I_dcr_* and *U_dci_*, *I_dci_* are represented as the DC voltage and current of the LCC and VSC stations, respectively; *X_smr_* and *X_smi_* are marked as the smoothing reactors installed at the LCC and VSC stations, respectively; *C_VSC_* is the DC capacitor of the VSC station.

It is assumed that the fault resistance is *R_g_* and the residual voltage at the fault location is *U_g_*. Herein, *R_dcr_*, *X_dcr_*, and *R_dci_*, *X_dci_* are represented as the resistance and reactance of the DC line of the LCC and VSC stations, respectively. As the SFCL resistance *R_SFCLr_* is connected in series with the LCC station, the dynamics of the DC fault current *I_dcr-f_* can be expressed as:

(2) $$\left(L_{smr}+L_{dcr}){\dot{I}}_{dcr-f}=U_{dcr}-U_{g}-{\overset{}{I}}_{dcr-f}(R_{SFCLr}+R_{dcr}\right)$$

If the DC voltage *U_dcr_* is represented by the function of firing angle, AC voltage and transformer leakage reactance, Equation (2) will be rewritten as:

(3) $$\begin{array}{cc} \{\; & \begin{array}{l} \left(L_{smr}+L_{dcr}){\dot{I}}_{dcr-f}=U_{dcr}-U_{g}-{\overset{}{I}}_{dcr-f}(R_{SFCLr}+R_{dcr}\right)\\ U_{dcr}=2(\frac{3\sqrt{2}V_{LCCm}}{\pi T}cos\alpha -{\overset{}{I}}_{dcr-f}\frac{3X_{T1}}{\pi }) \end{array}\\ \Rightarrow \; & (L_{smr}+L_{dcr}){\dot{I}}_{dcr-f}=\frac{6\sqrt{2}U_{LCCm}}{\pi T}cos\alpha -U_{g}\\ & -{\overset{}{I}}_{dcr-f}(R_{SFCLr}+R_{dcr}+\frac{6X_{T1}}{\pi }) \end{array}$$

where *U_LCCm_* is the root-mean-square (RMS) AC voltage over the LCC station; *T* is the transformer turn-ratio. As compared to the case of without SFCL, introducing *R_SFCLr_* is able to increase the resistance of the DC circuit, and it is helpful to reduce the peak value of the fault current. Considering the function of the firing angle controller, the working status of the LCC station will be changed from the rectifying mode to the inverting mode. Thus, the DC fault current will be enforcedly down to zero.

Note that, the residual voltage *U_g_* in Equation (2) can be calculated by:

(4) $$U_{g}=R_{g}(I_{dcr-f}+I_{dci-f})$$

where *I_dci-f_* is the DC fault current of the VSC station. It can be inferred that, when the ground resistance *R_g_* is not equal to zero and has a relatively large resistance value, the DC fault current of the LCC station might be potentially affected by that of the VSC station.

By referring to [24,25,26], the fault process of a VSC-HVDC station has three stages, which are DC-link capacitor discharging (stage 1), diodes freewheeling (stage 2) and grid-side current feeding (stage 3), respectively. Before the DC voltage drops to zero, all the free-wheel diodes are blocked due to the reverse voltage, and thus the DC link will be insulated from the AC grid 2. In a sense, stage 1 (capacitor discharging) is the key stage for the SFCL to suppress the DC fault current and mitigate the DC voltage decline.

**Figure 4:**
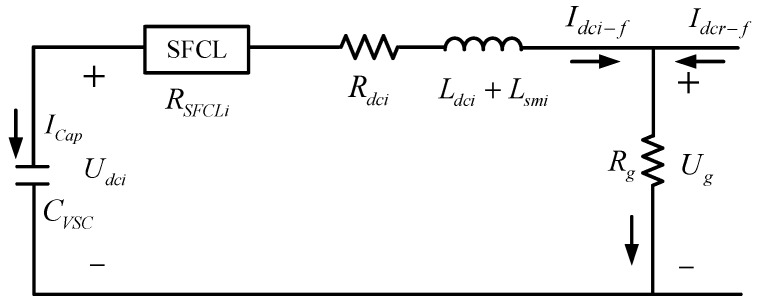
Fault analysis of the VSC-station with the SFCL (capacitor discharging stage).

Herein, stage 1 conducts the system response before the dc voltage drops to zero, and Figure 4 shows the fault analysis diagram. The circuit equation is modeled as:

(5) $$\begin{array}{cc} \{\; & \begin{array}{l} \left(L_{smr}+L_{dcr}){\dot{I}}_{dci-f}=U_{dci}-U_{g}-{\overset{}{I}}_{dci-f}(R_{SFCLi}+R_{dci}\right)\\ {\overset{}{I}}_{dci-f}=-I_{Cap}=-C_{VSC}{\dot{U}}_{dci} \end{array}\\ \Rightarrow \; & -(L_{smr}+L_{dcr})C_{VSC}{\overset{..}{U}}_{dci}=U_{dci}-U_{g}+C_{VSC}{\dot{U}}_{dci}(R_{SFCLi}+R_{dci})\\ \Rightarrow \; & (L_{smr}+L_{dcr})C_{VSC}{\overset{..}{U}}_{dci}+C_{VSC}{\dot{U}}_{dci}(R_{SFCLi}+R_{dci})+U_{dci}-U_{g}=0 \end{array}$$

By substituting Equation (4) into Equation (5), the equation will be rewritten as:

(6) $$\begin{array}{c} \left(L_{smr}+L_{dcr})C_{VSC}{\overset{..}{U}}_{dci}+C_{VSC}{\dot{U}}_{dci}(R_{SFCLi}+R_{dci}+R_{g}\right)\\ +U_{dci}-R_{g}I_{dcr-f}=0\; \end{array}$$

Regarding the solution method of Equation (6), details are analyzed in Appendix A.4. In theory, introducing *R_SFCLi_* will closely affect the VSC-HVDC link’s electrical properties, and it means that the current-limiting resistance *R_SFCLi_* can not only reduce the fault current level in the DC line, but also change the oscillation characteristic of the DC capacitor voltage. When increasing *R_SFCLi_* leads to an over-damped state, the capacitor voltage *U_dci_* will not decline to zero, and the subsequent two stages may not happen [27,28]. Owing to that all-diodes-conducting phenomenon is avoided, the SFCL’s contributions in reducing the currents in the AC side and the converter may become more obvious.

**Figure 5:**
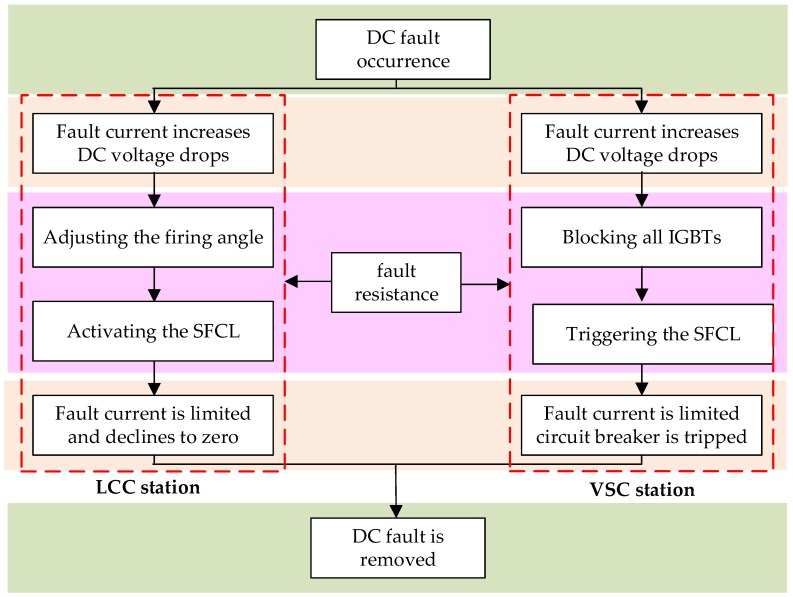
Flowchart of the integrated process of the proposed approach. IGBT: Insulated Gate Bipolar Translator.

According to the above theoretical analysis, the flowchart of the integrated process of the proposed approach can be shown in Figure 5.

## 3. Simulation Study

To evaluate the effectiveness of the SFCLs in the hybrid HVDC system, a detailed simulation model is built in the MATLAB software (R2017b, MathWorks, Natick, MA, USA), and the electromagnetic transient (EMT) type simulations are done in a 64-b personal computer with Intel i7-7700 QuadCore 2.8-GHz processor and 8-GB RAM (DELL, Round Rock, TX, USA). The EMT simulations use the discrete solver, and the simulation time step is set as 5 ×10^−5^ s.

The main parameters are summarized in Table 2, and the modeling information is depicted as: (i) The AC grid model is simulated by an AC voltage source in series with the equivalent resistance and inductance. (ii) The resistive SFCL model is based on the controlled voltage source [29]. (iii) The VSC and LCC adopt detailed models (detailed representation of power electronic converters), and the models are able to precisely show the dynamic performance over relatively short periods of times.

During the simulations, different SFCL resistances are taken into consideration [30,31], so as to validate how the change of the SFCL resistance affects the fault characteristics of the hybrid HVDC system. The estimated recovery time of the SFCL is about 4 s. For the resistance of *R_SFCL_* = 30 Ω, the coefficients of *a*_1_, *a*_2_, *b*_1_, *b*_2_ are set as *a*_1_ = 9.52, *a*_2_ = 15.87, *b*_1_ = 30, *b*_2_ = 19, respectively.

### 3.1. Changing the SFCL Resistance in the LCC Station

**Figure 6:**
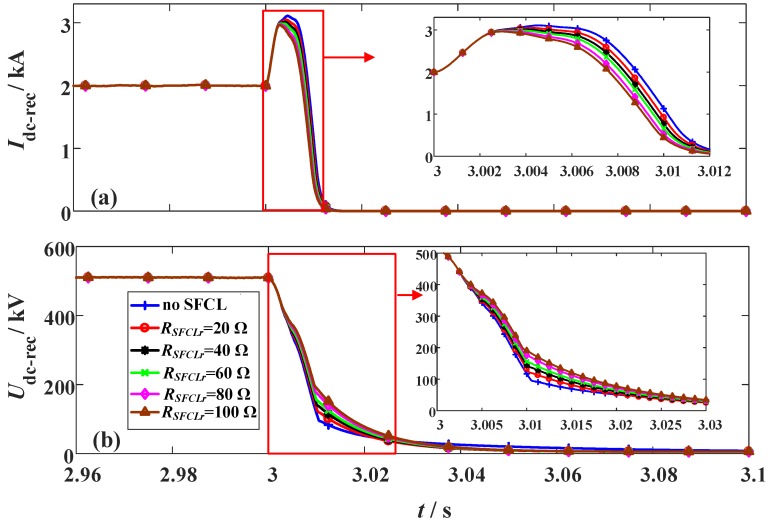
Behaviors of the LCC station considering the change of the SFCL resistance *R_SFCLr_*. (**a**) DC current and (**b**) DC voltage.

**Figure 7:**
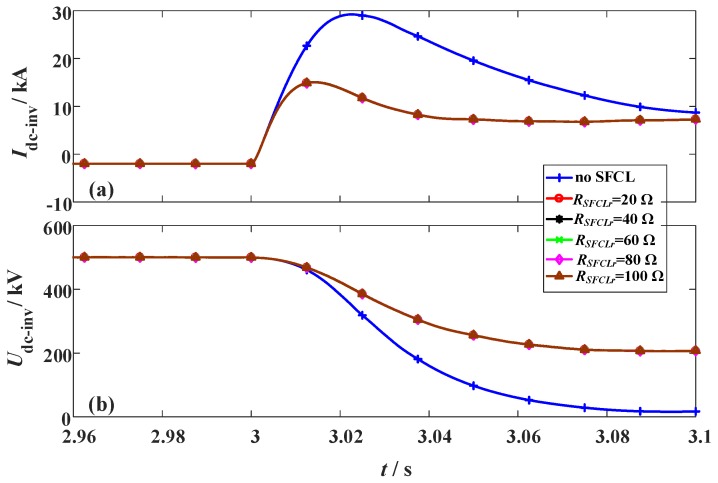
Behaviors of the VSC station considering the change of the SFCL resistance *R_SFCLr_*. (**a**) DC current and (**b**) DC voltage.

The simulation conditions of the DC short-circuit fault are defined as: (i) The fault occurs in the middle of the DC line at *t*_0_ = 3 s. (ii) The fault resistance and duration are 1 Ω and 100 ms. (iii) The SFCL at the LCC station (*R_SFCLr_*) changes from 20 Ω to 100 Ω, and the SFCL at the VSC station (*R_SFCLi_*) has the constant of 30 Ω. As shown in Figure 6 and Figure 7, they indicate the transient behaviors of the hybrid HVDC system subject to the change of *R_SFCLr_*.

In the LCC station, the peak value of the DC fault current is about 1.5 times of the rated level, and the reduction of the DC fault current is mainly conducted by adjusting the firing angle. Herein, the LCC station will switch to the inverting mode to make the DC fault current decline to zero. In the case of with the SFCL, it can mildly limit the fault current and alleviate the voltage drop. During the process of the fault feeding, the firing angle controller and the SFCL will serve as the primary and secondary factors to combinedly affect the fault transients.

It is observed that augmenting *R_SFCLr_* has almost no effect on the VSC station, where the activation of *R_SFCLi_* will undertake the crucial roles of current-limitation and voltage compensation. For the VSC station, installing the SFCL (*R_SFCLi_* = 30 Ω) is able to limit the DC fault current from 29.2 kA to 15.1 kA, and improve the DC voltage from 16 kV to 197 kV.

**Figure 8:**
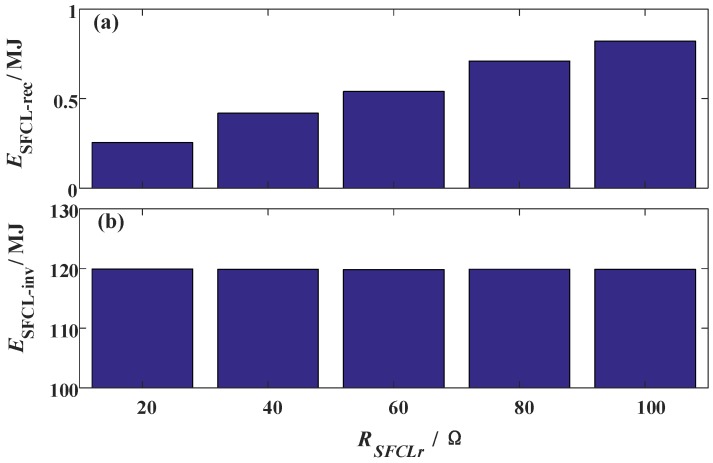
Energy dissipation of the two SFCLs subject to the change of the SFCL resistance *R_SFCLr_*.

Figure 8 shows the energy dissipation of the two SFCLs, where the calculation time is from the fault occurring to the fault being removed (the duration is 100 ms). When *R_SFCLr_* is designed as 20 Ω, 40 Ω, 60 Ω, 80 Ω, and 100 Ω, respectively, its dissipated energy at the LCC station will be 0.27 MJ, 0.44 MJ, 0.56 MJ, 0.73 MJ, and 0.85 MJ, respectively. A rising trend is obviously found, but the caused energy dissipation effect is still limitable. In the VSC station, its relevant SFCL has a steady and efficient energy dissipation with the level of 119.9 MJ.

### 3.2. Changing the SFCL Resistance in the VSC Station

In this subsection, the original fault parameters are unchanged, and it is designed that *R_SFCLr_* owns a constant of 20 Ω and *R_SFCLi_* varies from 10 Ω to 50 Ω.

**Figure 9:**
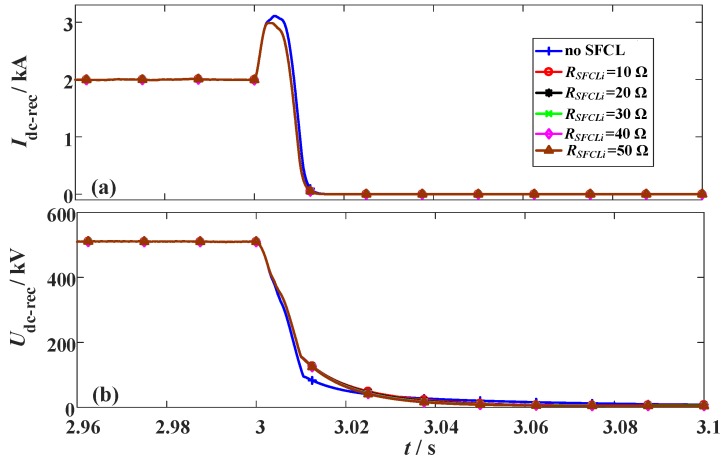
Behaviors of the LCC station considering the change of the SFCL resistance *R_SFCLi_*. (**a**) DC current and (**b**) DC voltage.

**Figure 10:**
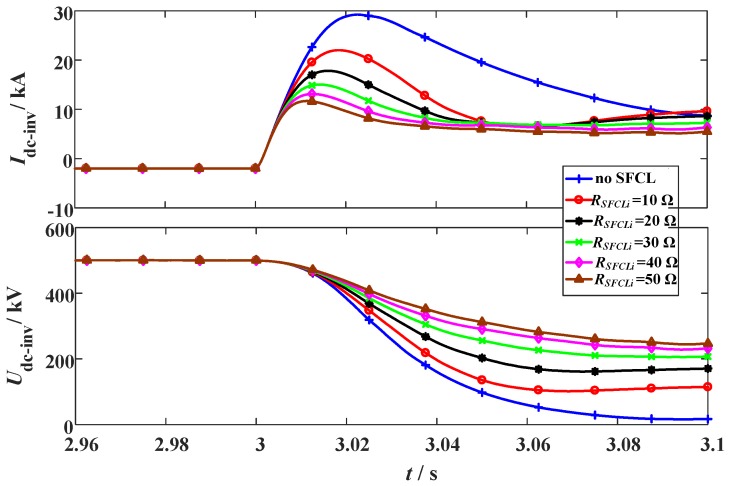
Behaviors of the VSC station considering the change of the SFCL resistance *R_SFCLi_*. (**a**) DC current and (**b**) DC voltage.

Figure 9 and Figure 10 show the characteristics of the hybrid HVDC system subject to the change of *R_SFCLi_*. According to the results, changing *R_SFCLi_* will obviously influence the transient fluctuations in the VSC station, but have a negligible effect on the DC current and voltage of the LCC station. In addition, it is found that a moderate increase of the SFCL resistance *R_SFCLi_* can bring better contributions. Nevertheless, it is not recommended to excessively increasing the resistance *R_SFCLi_*, since the current-limiting ratio of the SFCL seems to achieve the saturated level. When *R_SFCLi_* increases from 10 Ω to 20 Ω, the current-limiting ratio has an expected improvement of 14.3%, but when *R_SFCLi_* rises from 40 Ω to 50 Ω, the obtained improvement is just 4.8%. As shown in Table 3, it lists a detailed performance comparison.

**Figure 11:**
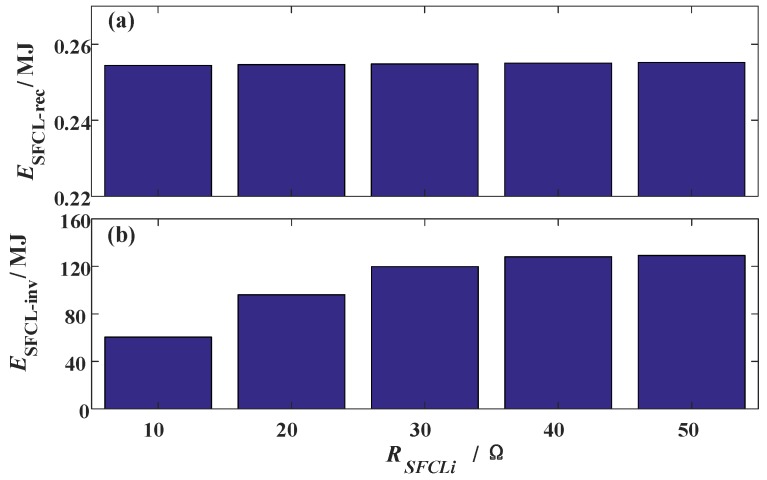
Energy dissipation of the two SFCLs subject to the change of the SFCL resistance *R_SFCLi_*.

Figure 11 shows the energy dissipation of the two SFCLs. Concerning that *R_SFCLi_* is set as 10 Ω, 20 Ω, 30 Ω, 40 Ω, and 50 Ω, respectively, the dissipated energy of the SFCL at the VSC station will be 60.5 MJ, 96.1 MJ, 119.9 MJ, 128.0 MJ, and 129.3 MJ, respectively. Regarding the LCC station, its relevant SFCL has a steady energy dissipation with the level of 0.27 MJ.

### 3.3. Changing the Fault Resistance of the Hybrid HVDC

**Figure 12:**
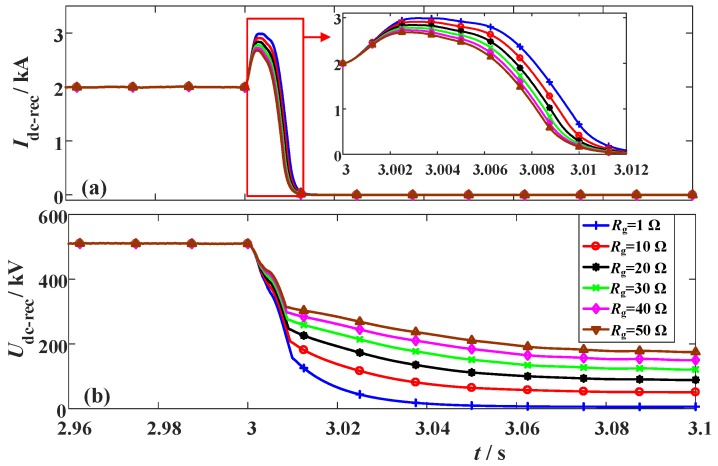
Properties of the LCC station considering the change of the fault resistance *R_g_*. (**a**) DC current and (**b**) DC voltage.

**Figure 13:**
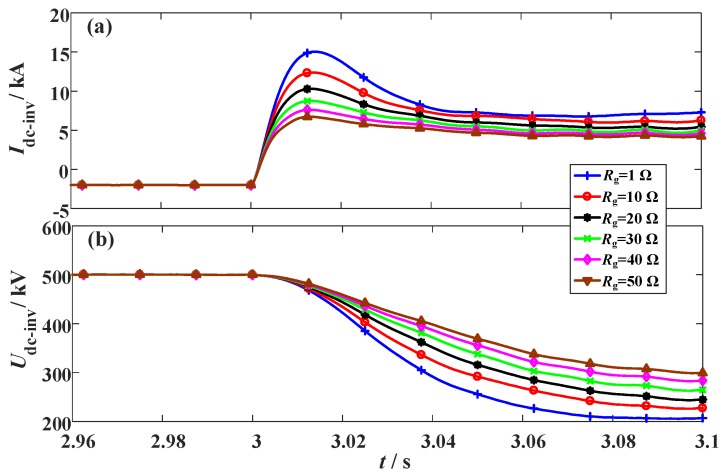
Properties of the VSC station considering the change of the fault resistance *R_g_*. (**a**) DC current and (**b**) DC voltage.

**Figure 14:**
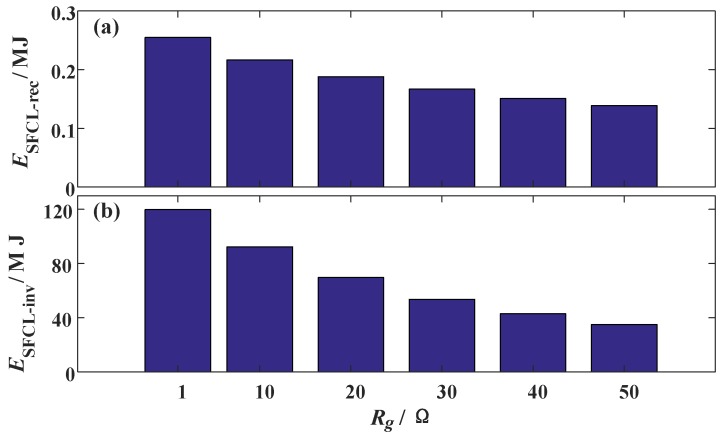
Energy dissipation of the two SFCLs subject to the change of the fault resistance *R*_g_.

As the fault resistance is a critical factor to evaluate the fault severity level and the behavioral interaction between the LCC and VSC stations, different fault resistances are simulated. The parameters of *R_SFCLr_* = 20 Ω and *R_SFCLi_* = 30 Ω are adopted, and the settings of the fault time and fault location are unchanged. The simulation waveforms are shown in Figure 12, Figure 13 and Figure 14.

From Figure 12, the main contribution of augmenting the fault resistance for the LCC station is to mitigate the DC voltage drop. The DC fault current still decreases to zero, but the DC voltage can be properly kept owing to the voltage support over the fault resistance. In the case of that the fault resistance is set as 10 Ω, 20 Ω, 30 Ω, 40 Ω, and 50 Ω, respectively, the DC voltage will reach to 50.8 kV, 88.6 kV, 119.2 kV, 143.1 kV, and 164.7 kV, respectively.

From Figure 13, the DC current and voltage of the VSC station will be both affected by the fault resistance, and a detailed performance comparison is given in Table 4.

Owing to the increase of the fault resistance, the dissipated energies in the two SFCLs are both reduced. Especially for the SFCL at the VSC station, an evident downswing is observed. For that *R_g_* is set as 10 Ω, 20 Ω, 30 Ω, 40 Ω, and 50 Ω, respectively, the dissipated energy of the SFCL at the VSC station will be 92.3 MJ, 69.7 MJ, 53.6 MJ, 42.9 MJ, and 34.9 MJ, respectively.

### 3.4. Changing the Fault Location of the Hybrid HVDC

**Figure 15:**
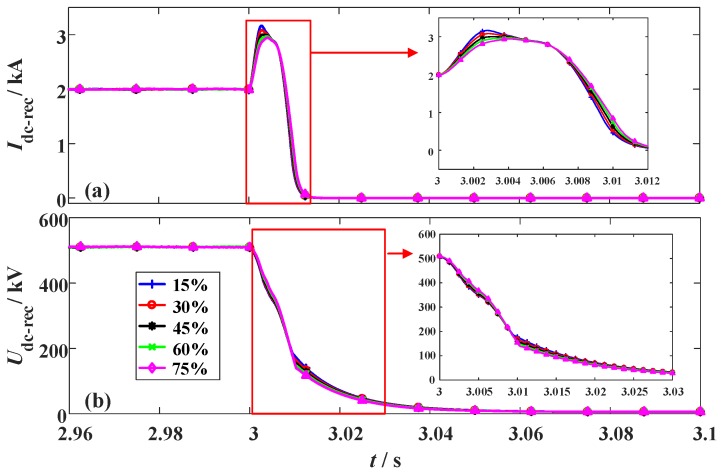
Simulation waveforms of the LCC station considering the change of the fault location. (**a**) DC current and (**b**) DC voltage.

**Figure 16:**
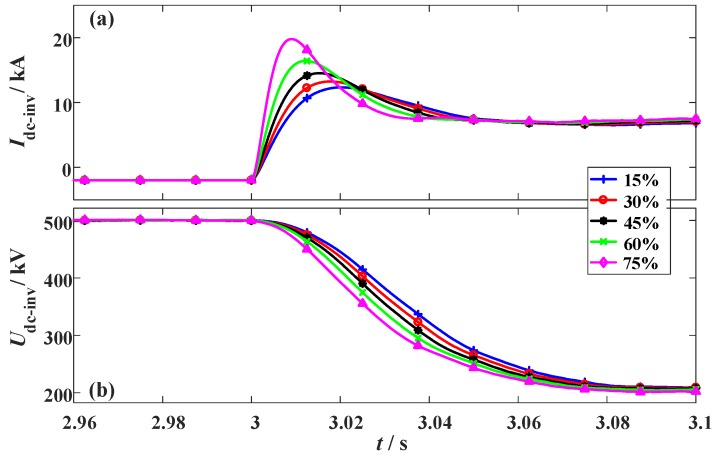
Simulation waveforms of the VSC station considering the change of the fault location. (**a**) DC current and (**b**) DC voltage.

**Figure 17:**
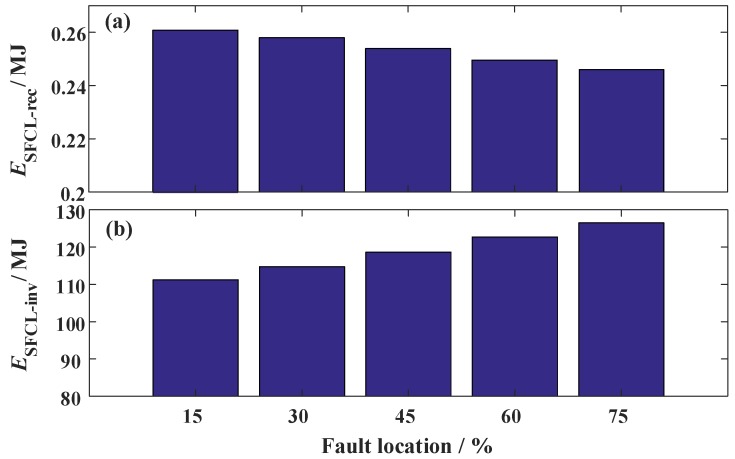
Energy dissipation of the two SFCLs subject to the change of the fault location.

To analyze how the fault location could affect the performance of the SFCLs, different fault sites are simulated. The fault location ratio is used to describe the relative position of the fault site in the whole DC line. When the fault location ratio increases, it means the fault site is farther away from the LCC station and closer to the VSC station. The two SFCLs still adopt *R_SFCLr_* = 20 Ω and *R_SFCLi_* = 30 Ω; the fault time and fault resistance are set as *t_0_* = 3 s and *R_g_* = 1 Ω, respectively. The simulation waveforms are shown in Figure 15, Figure 16 and Figure 17.

When the fault location ratio changes from 15% to 75%, the peak value of the DC fault current in the LCC station just changes from 3.11 kA to 2.97 kA. Considering that the expected reduction is just 0.14 kA, the SFCL at the LCC station has a low sensitivity to the fault location. In comparison, the DC fault current of the VSC station is more sensitive to the fault site. When the fault location ratio is 15%, 30%, 45%, 60%, and 75%, respectively, the corresponding current-limiting ratio will be about 44.9%, 46.5%, 47.9%, 49.3%, and 50.4%, respectively. It is proven that the SFCL at the VSC station has an enhanced current-limiting ability for handling the short-line faults.

Based on Figure 17, Table 5 shows the simulation data of the two SFCLs’ energy dissipation, whose changing trends are opposite with each other.

## 4. Scheme Design

**Figure 18:**
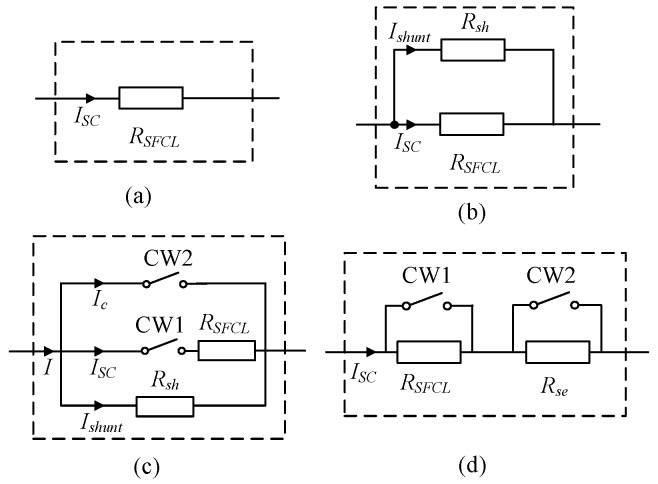
Candidates for the structure of the resistive SFCL used in the HVDC networks. (**a**) Pure resistive SFCL; (**b**) Resistive SFCL with external resistor in parallel; (**c**) Resistive SFCL with switches and external resistor in parallel; (**d**) Resistive SFCL with external resistor in series.

In this section, the SFCL scheme design is conducted. Firstly, the candidates for the structure of the resistive SFCL used in the HVDC networks are discussed. Figure 18a shows a general structure, which represents a pure resistive SFCL without an external resistor in parallel. Some scholars have applied this structure in [15,18], where the scholars consider the coordination of a high-speed direct-current circuit breaker (DCCB) and the SFCL. As the DCCB cuts off the DC fault current within 2–5 ms, the current-limiting time of the resistive SFCL can be controlled as 20 ms–50 ms. In light of a relatively short current-limiting time, the quench heat dissipation could be acceptable to a certain extent.

Figure 18b,c show two possible structures for the resistive SFCL with an external resistor in parallel [13,16,32]. In a sense, the scholars adopt a conservative and safe method, and the objective of introducing the external resistor is to avoid that the recovery process of the SFCL is too long. In addition, Figure 18d shows the structure of the resistive SFCL with an external resistor in series. The rating of the external resistor is the same as that of the resistive SFCL. When CW1 is closed and CW2 is opened, the external resistor will replace the SFCL to mitigate the fault transients. Hence, the current-limiting time of the resistive SFCL can be flexibly adjusted to ensure the safety and reliability of superconducting materials.

In this study, our research group prefers to use the general structure in Figure 18a. In case of this structure does not fully meet the requirement that the recovery time of the SFCL is about 4 s, the structure in Figure 18d can be regarded as an alternative solution. It should be noted that, the alternative structure may have the same current-limiting resistance as the preferred structure, and it does not affect the above simulation results of the DC current and voltage. In the following, the parameter selection is discussed.

For the LCC station, this study suggests installing the resistive SFCL with a lower quench resistance (no more than 20 Ω). On the one hand, it may cooperate with the firing angle controller to combinedly handle the DC fault issue. On the other hand, it may assist the SFCL at the VSC station to more powerfully handle the AC fault when the fault location is near the AC grid 1.

**Figure 19:**
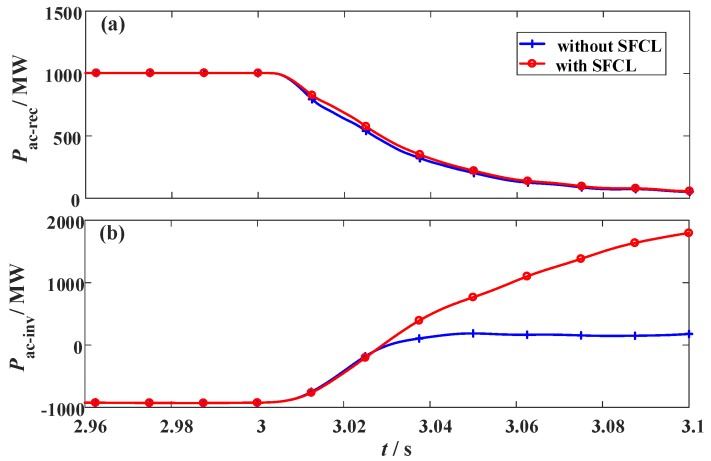
Power response of the AC systems under the fault. (**a**) AC grid 1 and (**b**) AC grid 2.

For the VSC station, this study recommends applying the resistive SFCL with *R_SFCLi_* = 30 Ω, which is sufficient to alleviate the DC voltage-current fluctuations and dissipate the active power. As shown in Figure 19, the power response of the AC systems is demonstrated, and here the fault resistance is *R_g_* = 1 Ω; the fault location is the middle of the DC transmission line.

Note that, it is not suggested to augment the SFCL resistance in excess. There might be a critical resistance value to depict the tradeoff among the SFCL cost, the fault current reduction and the inhibition of the voltage fluctuation [33]. Since detailed optimization and calculation are out of the scope of this paper, and will be presented in another report, a reasonable choice of *R_SFCLi_* = 30 Ω is adopted to implement the SFCL’s scheme design.

On basis of [34,35], a non-inductive unit coil for the SFCL is designed, and the coil parameters are listed in Table 6. To construct the SFCL at the VSC station, the normal current in the SFCL is 2 kA, and thus 15 pieces of coils connected in parallel are served as a coil group, which can meet the requirements of current capacity and safety margin. Further, 160 coil-groups connected in series is to obtain the quench resistance of 30 Ω.

## 5. Conclusions

In this paper, the application feasibility of resistive-type superconducting fault current limiters in a hybrid high voltage direct current transmission system is verified. The main conclusions are as follows:

1. The superconducting fault current limiter at the voltage source converter station enables to very efficiently mitigate the fault transients, and owns an enhanced current-limiting ability for handling the short-line faults. A moderate increase of the current-limiting resistance can bring better contributions, but an excessive increase may make the current-limiting ratio come up to the saturated level.
2. The superconducting fault current limiter at the line commutated converter station is able to mildly limit the fault current and alleviate the voltage drop, and its working performance has a low sensitivity to the fault location. As for the primary and secondary factors, the firing angle controller and the superconducting fault current limiter will combinedly handle the fault transients.

Concerning our future tasks, the optimization design, and economic evaluation of the superconducting fault current limiters will be done. Besides, the effects of the superconducting fault current limiters on the integration of large-scale renewable power sources into the hybrid system will be explored. These mentioned studies will be presented in the follow-up reports.

## Figures and Tables

**Table 1:** Summary of the studies of superconducting fault current limiters (SFCLs) in different high voltage direct voltage (HVDC) networks. LCC: line commutated converter; VSC: voltage source converter.

Type of SFCL	Type of HVDC	Voltage Class	Research Object	EvaluationMethod	Country, Report Year
Flux-coupling-type	LCC HVDC	230 kV	Commutation failure and fault recovery	MATLAB Simulation	China, 2015 [11]
Resistive type	LCC HVDC	180 kV [12] 500 kV [13]	Commutation failure and position analysis	PSCAD Simulation	Korea, 2016 [12] China, 2017 [13]
Hybrid-type	VSC HVDC	160 kV	Principle verification and scheme design	MATLAB Simulation	China, 2017 [14]
Resistive type	VSC HVDC	320 kV [15,19] 100 kV [16] 200 kV [18]	Techno-economic evolution and resistance varying behaviors	PSCAD [15,19] MATLAB [16,18]	France, 2017 [15] Korea, 2018 [16] China, 2017 [18] UAE, 2017 [19]
Inductive type	VSC HVDC	100 kV	Current limitation and recovery	MATLAB Simulation	Korea, 2018 [16]
Saturated iron-core-type	VSC HVDC	100 kV	Modeling, voltage analysis and energy dissipation	MATLAB Simulation	Korea, 2018 [16] China, 2018 [17]

**Table 2:** Main parameters of the simulation model.

Superconducting Fault Current Limiters	
Superconducting coil *R*_sc_ at the LCC/VSC	20 Ω–100 Ω/10 Ω–50 Ω
**LCC Station**	
Rated voltage/frequency	380 kV/50 Hz
Short-circuit ratio	3.076
DC current controller	*K _pIdc_* = 1, *K_iIdc_* = 90
**DC Link**	
Rated voltage/current	500kV/2 kA
Length of DC transmission line	500 km
Smoothing reactor of LCC/VSC	0.3 H/0.01 H
**VSC Station**	
Rated voltage/frequency	220 kV/50 Hz
Short-circuit ratio	3.34
AC current controller (*K_pVac_*, *K_iVac_*)	*K_pVac_* = 0.6, *K_iVac_* = 10
DC voltage controller (*K_pVdc_*, *K_iVdc_*)	*K_pVac_* = 8, *K_iVac_* = 20

**Table 3:** Performance of the SFCL at the VSC station under different parameters.

Items	Effects of the SFCL on the VSC Station	
	DC Fault Current/Current-Limiting Ratio	DC Voltage/Calculated Drop Rate
*R_SFCLi_* = 10 Ω	22.1 kA/24.7%	101.5 kV/79.7%
*R_SFCLi_* = 20 Ω	17.8 kA/39%	160.1 kV/67.9%
*R_SFCLi_* = 30 Ω	15.1 kA/48.5%	196.9 kV/60.6%
*R_SFCLi_* = 40 Ω	13.2 kA/55.1%	223.8 kV/55.2%
*R_SFCLi_* = 50 Ω	11.7 kA/59.9%	241.3 kV/51.7%

**Table 4:** Influence of the fault resistance on the VSC station.

Items	DC Fault Current		DC Voltage	
	No SFCL	With SFCL/Current-Limiting Ratio	No SFCL	With SFCL
*R_g_* = 10 Ω	21.2 kA	12.4 kA/41.7%	99.1 kV	222.2 kV
*R_g_* = 20 Ω	16.1 kA	10.3 kA/36.1%	159.1 kV	239.8 kV
*R_g_* = 30 Ω	12.8 kA	8.76 kA/31.7%	196.9 kV	252.1 kV
*R_g_* = 40 Ω	10.6 kA	7.62 kA/28.3 %	221.5 kV	261.8 kV
*R_g_* = 50 Ω	9.03 kA	6.74 kA/25.4%	239.9 kV	271.9 kV

**Table 5:** Influence of the fault location on the energy dissipation of the two SFCLs.

Fault Location Ratio	Energy Dissipation	
	SFCL at the LCC Station	SFCL at the VSC Station
15%	0.265 MJ	111.2 MJ
30%	0.258 MJ	114.8 MJ
45%	0.252 MJ	118.7 MJ
60%	0.247 MJ	122.7 MJ
75%	0.243 MJ	126.5 MJ

**Table 6:** Parameters of a non-inductive coil unit.

Parameter	Value
Length of YBCO tape (m)	54
Inner diameter (mm)	200
Outer diameter (mm)	870
Interturn gap (mm)	5
Resistance (Ω)	1.28 Ω @ 100 K
Resistance (Ω)	2.98 Ω @ 300 K
N Value (μV/cm)	38.6
Rated voltage (kV)	3
Break-down voltage (kV)	15
Rated current (A)	200
Peak current (A, with a duration of 100 ms)	900
Safety temperature limit (K)	300

**Figure A1:**
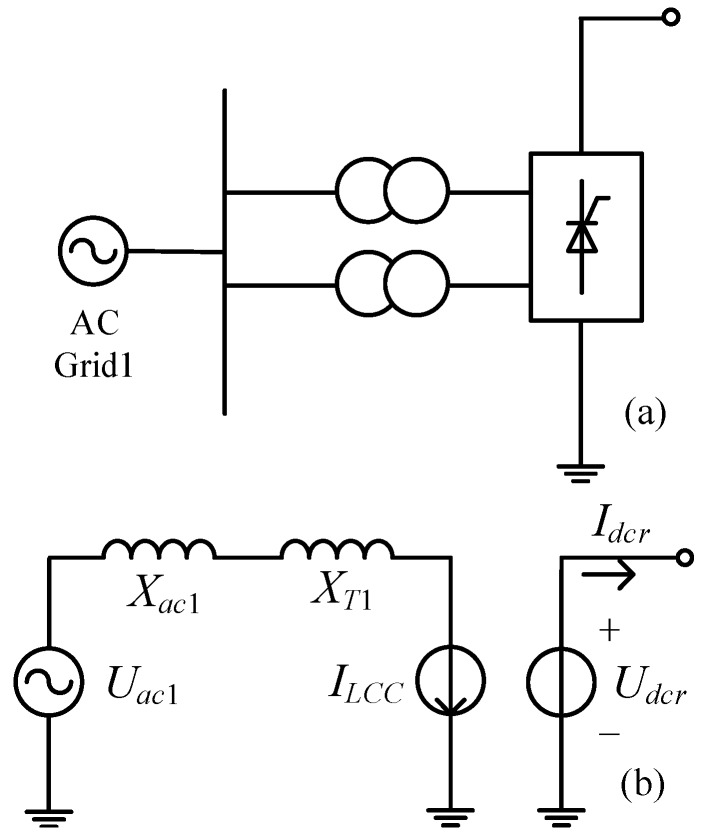
LCC station. (**a**) Connection diagram, (**b**) equivalent circuit.

**Figure A2:**
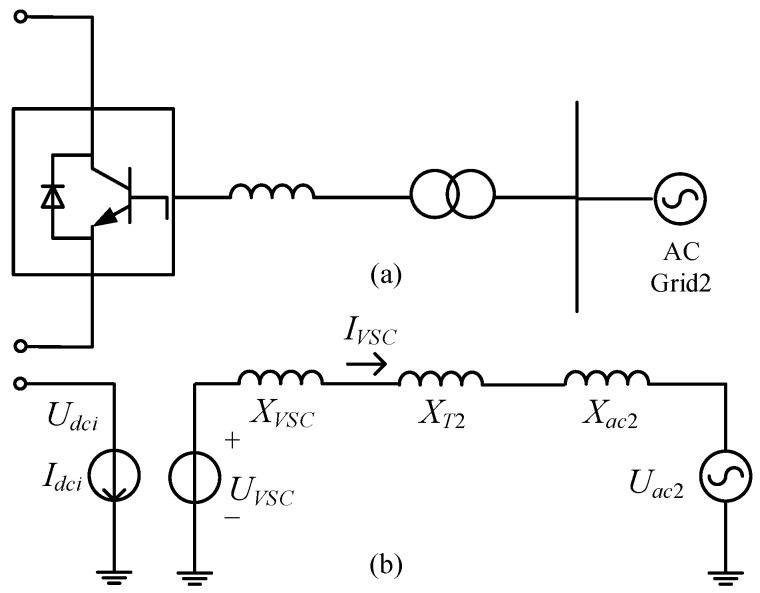
VSC station. (**a**) Connection diagram, (**b**) equivalent circuit.

**Figure A3:**
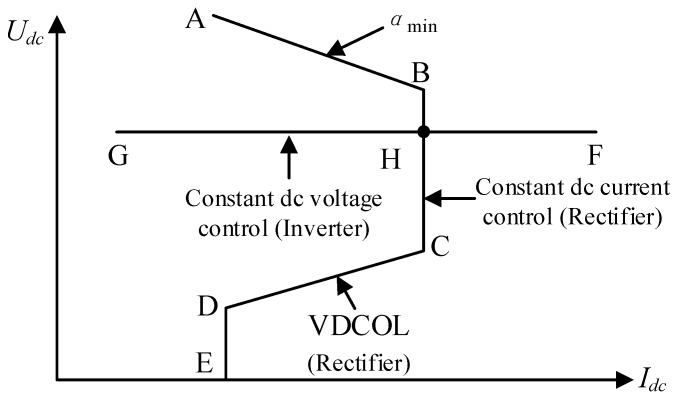
Basic control curves of the hybrid HVDC system.

## Nomenclature

LCC	Line commutated converter
VSC	Voltage source converter
RMS	Root mean square
FRT	Fault ride-through
HVDC	High voltage direct current
SFCL	Superconducting fault current limiter
YBCO	Yttrium barium copper oxide
VOCOL	Voltage-dependent current order limiter

## Symbols

*I*	Current [A]	*R*	Resistance [Ω]
*X*	Reactance [Ω]	*U*	Voltage [V]
*P*	Power [W]	*α*	Firing angle [Degree]
*M*	Modulation ratio [-]	*L*	Inductance [H]
*C*	Capacitance [F]		

## Subscripts

*c*	Commutation	*f*	Fault
*T*	Transformer	*r*	Rectifier side
*i*	Inverter side	*g*	Ground
*ac*	Alternating current	*dc*	Direct current
*sm*	Smoothing reactor	*min*	Minimum

## Appendix A

### Appendix A.1. LCC Station Modeling

Figure A1 shows the connection diagram and equivalent circuit of the LCC station. *U_ac_*_1_ and *X_ac_*_1_ are the equivalent voltage and reactance of the AC grid coupled to the LCC station, respectively; *X_T_*_1_ is the transformer leakage reactance; *I_LCC_* and *U_dcr_* indicate the equivalent AC current and DC voltage sources of the LCC station.

The switching functions of the AC current and the DC voltage are *S_LCC-abci_* (*S_LCC-ai_*, *S_LCC-bi_, S_LCC-ci_*) and *S_LCC-abcu_* (*S_LCC-au_*, *S_LCC-bu_, S_LCC-cu_*), respectively. The mathematical equations are obtained as follows:

(A1) $$\left[\begin{array}{c} i_{LCC-a}\\ i_{LCC-b}\\ i_{LCC-c} \end{array}\right]=\left[\begin{array}{c} S_{LCC-ai}\\ S_{LCC-bi}\\ S_{LCC-ci} \end{array}\right]I_{dcr}$$

(A2) $$\left[\begin{array}{c} U_{LCC-a}\\ U_{LCC-b}\\ U_{LCC-c} \end{array}\right]={\left[\begin{array}{ccc} S_{LCC-au} & S_{LCC-bu} & S_{LCC-cu} \end{array}\right]}^{-1}U_{dcr}$$

where *I_dcr_* is the DC current of the LCC station; *U_LCC_* is the voltage over the equivalent AC current source.

### Appendix A.2. VSC Station Modeling

Figure A2 shows the connection diagram and equivalent circuit of the VSC station. *U_ac_*_2_ and *X_ac2_* are the equivalent voltage and reactance of the AC grid coupled to the VSC station, respectively; *X_T2_* is the leakage reactance of the converter transformer; *X_VSC_* is the series reactance of the VSC; *U_VSC_* and *I_dci_* are the equivalent AC voltage and DC current sources of the VSC station.

The equations of voltage and current can be modeled as:

(A3) $$\left[\begin{array}{c} U_{VSC-a}\\ U_{VSC-b}\\ U_{VSC-c} \end{array}\right]=\left[\begin{array}{c} S_{VSC-au}\\ S_{VSC-bu}\\ S_{VSC-cu} \end{array}\right]U_{dci}$$

(A4) $$\left[\begin{array}{c} I_{VSC-a}\\ I_{VSC-b}\\ I_{VSC-c} \end{array}\right]={\left[\begin{array}{ccc} S_{VSC-ai} & S_{VSC-bi} & S_{VSC-ci} \end{array}\right]}^{-1}I_{dci}$$

### Appendix A.3. Control Modeling

Figure A3 shows the basic control curves of the hybrid HVDC system. For the LCC station (rectifier side), it configures a constant dc current controller and a voltage-dependent current order limiter (VDCOL). For the VSC station (inverter side), it adopts a direct current control mode, including an outer-loop voltage/reactive power controller and an inner-loop current controller.

### Appendix A.4. The solution of the DC Fault Current in the VSC Station

To seek a solution of the DC fault current in the VSC station, two assumptions are applied:

(i) Once the firing angle controller of the LCC station is activated, the DC fault current of the LCC station can be down to zero in a relatively short period after the fault;

(ii) The fault resistance is very little.

Supposing that the initial DC voltage and current are respectively marked as *U*_0_ and *I*_0_, the following equations are obtained:

(A5) $$U_{dci}=A_{1}e^{\lambda_{1}t}+A_{2}e^{\lambda_{2}t}$$

(A6) $$I_{dci-f}^{}=C_{VSC}(A_{1}\lambda_{1}e^{\lambda_{1}t}+A_{2}\lambda_{2}e^{\lambda_{2}t})$$

where *λ*_1_, *λ*_2_, *A*_1_, *A*_2_ are expressed as:

(A7) $$\begin{array}{cc} \lambda_{1,2}= & -\frac{R_{SFCLi}+R_{dci}+R_{g}}{2(L_{smr}+L_{dcr})}\\ & \pm \sqrt{{\left(\frac{R_{SFCLi}+R_{dci}+R_{g}}{2(L_{smr}+L_{dcr})}\right)}^{2}-\frac{1}{(L_{smr}+L_{dcr})C_{VSC}}} \end{array}$$

(A8) $$\left\{ \begin{array}{l} A_{1}=\frac{\lambda_{2}U_{0}+I_{0}/C_{VSC}}{\lambda_{2}-\lambda_{1}}\\ A_{2}=\frac{\lambda_{1}U_{0}+I_{0}/C_{VSC}}{\lambda_{1}-\lambda_{2}} \end{array}\right.$$
